# Semantic Verbal Fluency test in dementia: Preliminary retrospective analysis

**DOI:** 10.1590/S1980-57642009DN30400009

**Published:** 2009

**Authors:** Marcos Lopes, Sonia Maria Dozzi Brucki, Viviana Giampaoli, Letícia Lessa Mansur

**Affiliations:** 1 PhD, Department of Linguistics, Faculty of Philosophy, Letters and Humanities of the University of São Paulo, São Paulo SP, Brazil.; 2 MD, PhD, Behavioral and Cognitive Neurology Unit, Department of Neurology of the University of São Paulo School of Medicine and Cognitive Disorders Reference Center (CEREDIC), Hospital das Clínicas of the University of São Paulo School of Medicine, and Hospital Santa Marcelina, São Paulo SP, Brazil.; 3 PhD. Department of Statistics – Mathematics and Statistics Institute of University of São Paulo, São Paulo SP, Brazil.; 4 PhD. Department of Physiotherapy, Speech-Pathology and Occupational Therapy of Medicine School of University of São Paulo, São Paulo SP, Brazil.

**Keywords:** verbal fluency, evaluation, cognition, dementia

## Abstract

The Semantic Verbal Fluency (SVF) test entails the generation of words from a given category within a pre-set time of 60 seconds.

**Objectives:**

To verify whether socio-demographic and clinical data of individuals with dementia correlate with the performance on the SVF test and to ascertain whether differences among the criteria of number of answers, clusters and data spread over the intervals, predict clinical results.

**Methods:**

This was a retrospective study of 49 charts of demented patients classified according to the Clinical Dementia Rating (CDR) scale. We correlated education, age and gender, as well as CDR and Mini-Mental State Exam (MMSE) scores with the number of answers, clustering and switching distributed over four 15-second intervals on the SVF test.

**Results:**

The correlation between number of answers and quartiles was weak (r=0.407, p=0.004; r=0.484, p< 0.001) but correlation between the number of clusters and responses was strong (r=0.883, p< 0.001). The number of items on the SVF was statistically significant with MMSE score (p=0.01) and there was a tendency for significance on the CDR (p=0.06). The results indicated little activity regarding what we propose to call *cluster recalling* in the two groups.

**Discussion:**

The SVF test, using number of items generated, was found to be more effective than classic screening tests in terms of speed and ease of application in patients with CDR 2 and 3.

## Abstract (pt)

A Fluência Verbal Semântica (FVS) requer a geração de palavras de determinada categoria, num tempo pré-fixado de 60 segundos.

**Objetivos:**

Verificar se os dados sociodemográficos e clínicos de indivíduos com demências se correlacionam com a FVS; apontar possíveis diferenças entre critérios de número de respostas, agrupamentos e dados distribuídos nos intervalos predizem resultados clínicos.

**Métodos:**

Este é um estudo retrospectivo de 49 prontuários de pacientes com demência, classificados de acordo com a escala de estadiamento de demência (*Clinical Dementia Rating*-CDR). Foram correlacionados os dados de educação, idade e gênero e CDR e Mini-Exame do Estado Mental (MEEM) ao número de respostas, agrupamentos e mudanças de critério (switchings) gerados, em quatro intervalos de 15 segundos.

**Resultados:**

As correlações entre número de respostas, quartis e entre número de respostas e de quartis foram baixas (r=0.407, p=0.004; r=0.484, p< 0.001); porém, a correlação foi alta entre número de agrupamentos e de respostas (r=0.883, p< 0.001). O número de itens gerados na SVF foi estatisticamente significante com o MMSE (p=0.01) e houve tendência à significância no CDR (p=0.06). Os resultados indicaram pouca atividade daquilo que chamamos de *cluster recalling* nos dois grupos.

**Discussão:**

A FVS com o critério de itens gerados pode ser considerado instrumento clínico vantajoso em relação às varreduras clássicas, em pacientes com CDR 2 e 3, pela rapidez e facilidade de aplicação.

The clinical evaluation of semantic memory includes the verbal fluency test, in which an individual is required to recall items. Variations of this test include the phonemic verbal fluency (PVF), free fluency, fluency of certain classes of words, alternated fluency, and semantic verbal fluency (SVF) of different semantic categories such as animals, food, fruits and supermarket items. Generally, one-minute recuperation time is allowed during tests. The SVF test is a quick, easy-to-apply test which presents high sensitivity and specificity for the diagnosis of dementia, justifying its use to detect cognitive decline, either applied individually or in cognitive evaluation batteries such as the Consortium to Establish a Registry for Alzheimer’s disease (CERAD),^6^ Mattis Dementia Rating Scale (DRS)^4,5^ and Brief Cognitive Screening Battery (BCSB).^7^

Many authors have reported that age had greater impact on SVF while schooling had no influence on the PVF.^1,2,4-6,8^

With regard to lesions and cognitive repercussions, recent review of the literature on the use of VF in evaluations of patients with focal cortical lesions concluded that semantic verbal fluency related to animals was more specific in detecting cognitive alterations resulting from temporal lesions, while PVF more accurately detected deficits resulting from frontal lesions. Prior studies using functional magnetic resonance in focal lesions, have highlighted the implication of bilateral pre-frontal and dorso-lateral cortices and ventral median areas in SVF.^9,10^ Most studies investigating demented performance on SVF tests have been involved Alzheimer’s subjects.^11^ In Alzheimer’s disease (AD), there is progressive disturbance of semantic memory, attributed to alterations in the inferior-lateral temporal and frontal lobes.^12^

Other types of dementia also compromise performance in tasks of semantic information recall. Not only is verbal fluency sensitive for the detection of cognitive alterations, it also aids in the differential diagnoses of AD, vascular dementia (VD), mild cognitive impairment and is also useful in follow-up and establishing degree of compromise.^13-15^ Furthermore, it has assisted in predicting the course of the disease and survival.^16^

SVF can be applied using different methods. The most frequent is tracking the number of items uttered, according to certain semantic or phonemic criteria, within a pre-set time. Some studies have sought to sensitize fluency analysis by introducing methods that qualitatively analyze clusters and switching, which has proved productive in evaluating patients with various sub-type dementias. There is however disagreement about clusters and switching and consequently over scoring methods used in the analysis of fluency results.

Troyer^17^ proposed analyzing sub-category (clusters) and the capacity of changing to a new category when a sub-category has been exhaustively explored (switching). According to the author, clustering is linked to the temporal lobe and depends on verbal memory and retrieval of verbal stock. A decrease in clusters is typical in AD and other temporal lobe diseases. Troyer also defends the idea that switching requires strategic skills, such as cognitive flexibility and a change of mental setting, and is related to executive or pre-frontal dysfunction, such as those that occur in Parkinson’s or Huntington’s disease. The criteria for the constitution of semantic category for cluster analysis, according to Troyer, encompasses the overlapping of formal (semantic traces of class formation) and functional (as in the idea of “pets”) criteria and even the overlapping of categories. Numerous theoretical approaches exist that are psychologically,^18^ linguistically^19^ or neurolinguistically^20^ based and which support the hypothesis of semantic class conceptual blending. Sophisticated methods propose the analysis of internal changes among the item groupings,^21^ which are more related to generating items under pressure, being characteristic of the task.

Our objective was (1) to compare the traditional analysis of verbal fluency which tracks the number of items uttered with an approach that analyzes clusters and switching, in a sample of CDR stage 1 and 2 patients; (2) to verify the predictive value of fluency for CDR functionality.

## Methods

### Patients

This was a retrospective study of 49 cases selected from patients evaluated at the Behavioral and Cognitive Neurology Unit of the Hospital das Clínicas in São Paulo. This public university hospital institution receives socially heterogeneous patients ranging from illiterates to graduates and unemployed to economically sound individuals.

Medical files of patients with dementia who had been submitted to the Mini-Mental State Examination (MMSE), Clinical Dementia Rating (CDR) and SVF test were investigated. Files of patients whose SVF scores had been registered for each 15 seconds of the 1-minute test were selected. Socio-demographic data are presented in Table 1, and MMSE and CDR scores are depicted in Table 2.

**Table 1:** Sociodemographic data.

CDR (Number of subjects)	Age mean (SD)	Schooling mean (SD)
CDR 1 (n=22)	70.14 (10.33)	3.91 (2.45)
CDR 2/3 (n=27)	71.52 (11.33)	5.37 (4.24)
Total (n=49)	70.90 (10.80)	4.71 (3.60)

CDR, Clinical Dementia Rating; SD, standard deviation

**Table 2:** MMSE and CDR scores, number of generated answers, clusters, cluster size and intervals on SVF.

CDR	MMSE	Number of answers Mean (SD)	Number of clusters Mean (SD)	Cluster size Mean (SD)	Number of intervals Mean (sd)
CDR 1	19.86 (4.15)	8.59 (4.19)	2.95 (1.70)	2.50 (1.70)	2.95 (1.70)
CDR 2/3	13.70 (4.33)	5.22 (3.19)	1.40 (1.27)	2.24 (1.92)	2.22 (1.28)
Both groups	16.46 (5.22)	6.73(4.00)	2.10(1.66)	2.36 (1.59)	2.53(1.17)

CDR, Clinical Dementia Rating; MMSE, Mini-mental State Examination; SD, standard deviation.

### Verbal fluency test

Results from the SVF test are normally validated and then classified according to some clustering criteria. In this study, an answer was considered invalid if it had already been mentioned (i.e., a repeated occurrence) or if it did not name an animal; all other answers were considered valid. Clustering criteria were based upon semantic classes of words. Groups of at least three such answers are commonly considered to form a cluster. In this study, we considered groups of two semantically related answers as a cluster.

We treated classification of animal entries differently to SVF procedures found in the traditional literature. First, we did not follow the academic zoological classification of animals (insects, mammals, etc.), but rather used much broader traits: *wild, domestic environment* (which includes pets such as cats and dogs, but also frogs, worms and mosquitoes, all easily found in a house garden), *breeding, small* (for arthropods and such like), *winged* and *aquatic animals*. Secondly, we considered classes as non-exclusive, meaning that a single animal may fall into many classes. A duck, for example, may be considered a wild or a breeding or a winged animal, depending on the context. The context in our study was simply the number of the animal semantic traits, which permitted clustering relationships provided other neighboring entries shared the same traits.

All classification, cluster formation and inferences on data treatment (prior to statistics) were done automatically by a VBA program on a Microsoft Access database. A results table was generated from patient IDs and contained all answers, all clusters, the distribution of answers over the four 15-second intervals, and some descriptive data (number of answers, number of clusters, etc.).

### Statistical analysis

Statistical analysis was performed using the *R Project* software.^22^ The significance value was fixed at 0.05 for clinical purposes, although a higher value was applied to verify tendencies.

Nonparametric tests were used to compare patient groups (Kruskall-Wallis test) and correlations were performed by Spearman’s correlation test.

## Results

Sociodemographic data from the medical files of the 49 selected cases are presented in Table 1, and MMSE and SVF scores are shown in Table 2.

Different types of dementia were included in the sample, as the main objective of the study was to explore the method of analyzing verbal fluency. In the two CDR groups, there was a predominance of thirty three AD patients while nine patients were diagnosed as VD and seven had other diagnoses (dementia post radiotherapy, normal pressure hydrocephalus, dementia syndrome plus ataxia, cortico basal degeneration, frontotemporal dementia, meningeal herpes encephalitis).

Given that the performance of subjects with CDR 2 was similar to CDR 3 individuals, we chose to pool these into a single group.

The time of manifestation of diseases varied between 1 and 10 years.

The correlation between the number of answers and number of intervals was low (r=0.407, p=0.004, r=0.484, p< 0.001). However, correlation was high between the number of clusters and the number of answers (r=0.883, p< 0.001). Correlations between mean cluster size and MMSE, as well as between mean cluster size and number of intervals was considered null (r=0.076, p=0.603; r=0.187, p=0.197). Correlation between number of answers and mean cluster size was low (r=0.456, p=0.001). Differences between the two CDR groups concerning the mean cluster size did not reach significance according to the Kruskall-Wallis test (p=0.531).

Considering the CDR classes, groups CDR1 and CDR2 demonstrated a high correlation between number of clusters and number of answers (0.877 and 0.825, p< 0.001, respectively).

The associations between the MMSE and independent variables were analyzed using a Poisson regression model, adjusted for age, gender, schooling, CDR and interactions. For the analysis of deviance, we concluded that there was no significant association between age and schooling and MMSE scores (p-values were greater than 0.20). In contrast, a significant main effect of gender (p=0.015) and CDR (p< 0.001) was detected on the MMSE.

Considering the characteristics of the data – scores compiled, the response variables, number of answers, number of clusters and number of intervals were modeled as a Poisson distribution. The Poisson regression model adjusted for age, gender, schooling and CDR was considered for each variable. All the simple effects and the interaction terms between schooling and CDR, and between MMSE and CDR were fitted. The Akaike Information Criterion (AIC) (268) for choosing the “adequate” model suggested that number of answers was MMSE (p=0.01) and CDR (p=0.06, which means tendency toward significance).

The expected values for the number of answers for different MMSE values are presented in Table 3 for this model. The model fit only with the predictor MMSE.

**Table 3:** Number of answers and MMSE.

MMSE	CDR=1	CDR=2/3	Excluding CDR
5	4.767	3.673	3.256
10	5.789	4.462	3.953
15	7.028	5.415	4.798
20	8.536	6.575	5.828
25	10.361	7.983	7.076
30	12.580	9.693	8.591

CDR, Clinical Dementia Rating; MMSE, Mini-Mental State Examination; SD, standard deviation.

We drew the same conclusion for the number of clusters with MMSE (p=0.06) and CDR (p=0.06) (AIC=170.7). There was a tendency toward significance in the main effect of MMSE (p=0.06) on number of intervals (AIC=162).

Results of the clusters and switching between the two CDR groups were compared using a CDR-adjusted logistic model (Table 4).

**Table 4:** Cluster recalling in CDR groups.

	Total	Non-recalling patients (%)	Recalling patients (n%)
CDR 1	22	16/49 (33%)	6/49 (12%)
CDR 2/3	27	24/49 (49%)	3/49 (6%)
Both groups	49	40 (82%)	9 (18%)

CDR, Clinical Dementia Rating.

Finally, we also analyzed data related to what we propose to call *cluster recalling*: after generating a number of answers forming different clusters, the patient (usually after a pause) produces a new answer belonging to a cluster which has already been formed. Typically, the recalled cluster is the first one generated, but the last one was found in some cases, thus closing the series of clusters. The observed results are summarized in Table 4. For this purpose, we considered a logistic model adjusted for CDR.

Results show that a minority of patients (18%) exhibit cluster recalling in both CDR groups. There was a tendency for statistically significant difference between the two groups adopting a 10% level (F-test; p=0.085). Estimated model probability for CDR 2 patients to present cluster recalling was 0.11 versus 0.32 for CDR 1 patients.

## Discussion

The objective of this study was to compare two methods of verbal fluency analyses in a sample of demented patients, with CDR 1 and 2/3.

In this study, we considered groups of two semantically related answers as a cluster on the basis that there is no sound rationale why three but not two conceptual entries should form a group, and because the abstract association linking three (or more) words was also valid for two words. Although it is always possible to conceive a single object as a member of a class, exactly which class it belongs to cannot be accurately determined unless such classes are considered to be “natural classes”, i.e., prior to application of cultural and linguistic criteria. Some authors^21^ consider isolated answers as a criterion to divide clusters.

The rationale for the decision not to adopt academic classification of animals is supported by the fact that people often classify animals according to their appearance and not their “natural” families (e.g., a *dolphin* would more readily associated with a *shark*, as the two live in the sea, than with a *horse*, although they are both mammals), and this is further accentuated in the case of illiterates. The decision to consider classes as non-exclusive (a single animal may fall into many classes) was supported by the number of animal semantic traits, which allows clustering relationships provided other neighboring entries share the same traits. This concept is congruent with the connectionist theory of a semantic information network organization.^20^ Naturally, this entails a potentially larger number of clusters than the exclusive classes method, and furthermore, it is relatively common to have a cluster of one class included in another (i.e. a three-wild-animal cluster in a five-breeding-animal one). In such cases, only the larger cluster is registered (because it likely that which best reflects the subject’s thought) and its “subcluster” is disregarded.

Since we have weakened the two criteria for cluster formation (reducing minimum cluster size to two and allowing animal semantic entries to fall under many classes), we might naturally expect to have higher scores in number of clusters as well as in cluster sizes which in turn tends to increase the correlation between the number of answers and the number of clusters. This might explain why many of the test scores were taken into account when checking their statistical dependence on CDR and MMSE.

Although a larger sample would be crucial to determine the scope and validity of these results, our findings have important implications for SVF evaluation procedures since the number of answers alone, for instance, seems to hold all the information needed for performing statistical analysis.

The association between gender and MMSE, although an interesting point warranting further exploratory studies, digressed from the scope of this study in as far as no gender differences were verified in the SVF indicators.

The fact that socio-demographic data did not directly statistically correlate with clinical score may be attributed to the variability of diseases included in the sample for these levels of CDR. Moreover, with the predominance of moderate-severe patients in this sample, it is possible that we crossed the cut-off point allowing disease to then predominate over the effects of age and schooling, a phenomenon found in previous studies on demented patrients.^23^

Considering that the SVF test is much easier to apply and evaluate than the CDR or the MMSE, and that it strongly correlates with the scores of these tests, it is noteworthy that the SVF scores could be statistically modeled for use in differential diagnoses, similar to the two other clinical exams.

With regard to cluster recalling analysis, the results indicate a tendency for a significant difference of 10% between the two CDR groups. If a significance level of 5% were considered, the groups would not present significant differences, but the reason for accepting a tendency of significance can be ascribed to the small sample size of each of the groups, principally when considering the generally low occurrence of switching. Therefore, it is to be expected that studies involving larger populations can confirm the tendency of these data and present significant differences of 5%. These differences observed between the groups likely stem from the fact that the activity of recalling mobilizes more resources than the simple emission of answers or their grouping into specific semantic categories.

The fact that the recalled semantic categories are more often the first one brought up by the patients is worthy of mention. This may indicate that semantic classes which are more immediately accessible (in the first answers) are more likely to remain in working memory while the patient mobilizes other classes. This recall may be interpreted as a situation whereby the subject had not exhausted their lexical competence on the class and most importantly, was able to select *other elements* within that class, i. e., new names, different to all that had previously been generated, which indicates patient ability to mobilize executive functions. This is confirmed by the superior performance of CDR 1 patients on cluster recalling. Furthermore, there seems to be some manner of association between the recalled categories and their prototypicality levels:^18,19^ the more prototypical a class is (such as *mammals*), the more frequently it is recalled; and the more prototypical a member of that class is (*e. g., dog, cat, horse...*), the more frequently it is cited as the first answer in that class.

In a sample of patients who were predominantly at moderate or severe stages, it is possible that dementia has compromised further substrate^11^ and, in addition to the difficulty of verbal stock retrieval, there is also executive dysfunction, which leads to strategic inefficiency.

From a clinical point of view, it is opportune to pose the question whether analysis of clusters and switching would be best indicated to analyze CDR1 patients, while its application among CDR2 and CDR3 groups would merely serve to yield information on their worsening condition.

Future studies are necessary to verify the clinical applicability of this method. Investigations should include a larger sample of subjects, grouped according to specific diagnoses and severity of functional compromise.

## Figures and Tables

**Figure 1:**
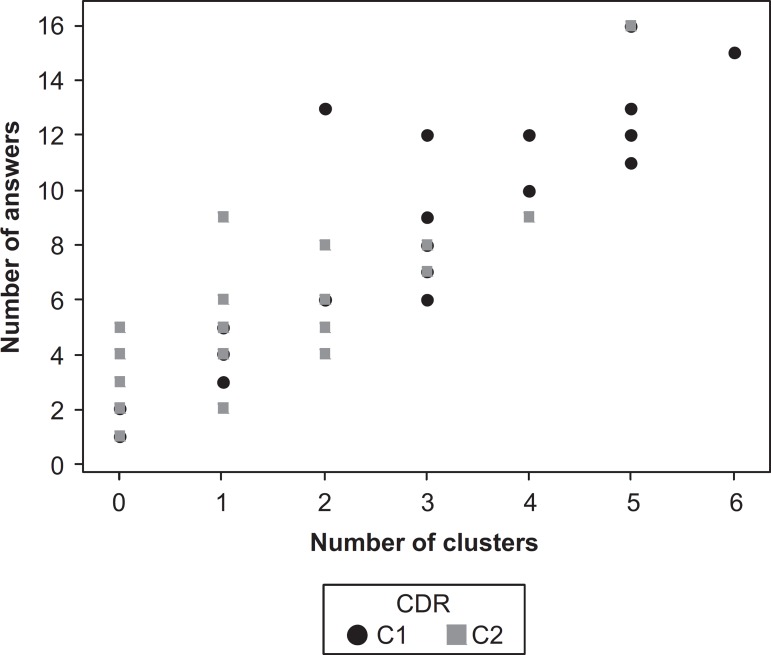
Correlation of number of clusters and number of answers.
